# Impact of growth matrix on pharmacodynamics of antimicrobial drugs for pig pneumonia pathogens

**DOI:** 10.1186/s12917-017-1086-4

**Published:** 2017-06-23

**Authors:** Lucy Dorey, Peter Lees

**Affiliations:** The Royal Veterinary College, Hawkshead Campus, Herts, AL97TA, Hatfield, UK

**Keywords:** Minimum inhibitory concentration, Florfenicol, Oxytetracycline, Marbofloxacin, *A. Pleuropneumoniae*, *P. Multocida*

## Abstract

**Background:**

The most widely used measure of potency of antimicrobial drugs is Minimum Inhibitory Concentration (MIC). MIC is usually determined under standardised conditions in broths formulated to optimise bacterial growth on a species-by-species basis. This ensures comparability of data between laboratories. However, differences in values of MIC may arise between broths of differing chemical composition and for some drug classes major differences occur between broths and biological fluids such as serum and inflammatory exudate. Such differences must be taken into account, when breakpoint PK/PD indices are derived and used to predict dosages for clinical use. There is therefore interest in comparing MIC values in several broths and, in particular, in comparing broth values with those generated in serum. For the pig pneumonia pathogens, *Actinobacillus pleuropneumoniae* and *Pasteurella multocida*, MICs were determined for three drugs, florfenicol, oxytetracycline and marbofloxacin, in five broths [Mueller Hinton Broth (MHB), cation-adjusted Mueller Hinton Broth (CAMHB), Columbia Broth supplemented with NAD (CB), Brain Heart Infusion Broth (BHI) and Tryptic Soy Broth (TSB)] and in pig serum.

**Results:**

For each drug, similar MIC values were obtained in all broths, with one exception, marbofloxacin having similar MICs for three broths and 4–5-fold higher MICs for two broths. In contrast, for both organisms, quantitative differences between broth and pig serum MICs were obtained after correction of MICs for drug binding to serum protein (fu serum MIC). Potency was greater (fu serum MIC lower) in serum than in broths for marbofloxacin and florfenicol for both organisms. For oxytetracycline fu serum:broth MIC ratios were 6.30:1 (*P. multocida*) and 0.35:1 (*A. pleuropneumoniae*), so that potency of this drug was reduced for the former species and increased for the latter species. The chemical composition of pig serum and broths was compared; major matrix differences in 14 constituents did not account for MIC differences. Bacterial growth rates were compared in broths and pig serum in the absence of drugs; it was concluded that broth/serum MIC differences might be due to differing growth rates in some but not all instances.

**Conclusions:**

For all organisms and all drugs investigated in this study, it is suggested that broth MICs should be adjusted by an appropriate scaling factor when used to determine pharmacokinetic/pharmacodynamic breakpoints for dosage prediction.

## Background

Dosage regimens of antimicrobial drugs should be optimized first to achieve clinical and bacteriological cure and second to provide minimal opportunities for the selection of resistant organisms. The successful outcome of therapy depends on both the pharmacokinetic (PK) profile, which determines the drug concentration-time course at the site of infection and the pharmacodynamic (PD) profile that is drug effect on the pathogen [1–4]. Hence, a rational approach to dose prediction, for subsequent confirmation or adaptation in disease models and clinical trials, is dependent on PK/PD integration and modelling methods. These establish PK/PD breakpoints for pre-determined levels of kill [2–6].

Minimum Inhibitory Concentration (MIC) is the primary PD parameter used to determine potency; it is the lowest drug concentration that inhibits visible bacterial growth after 16–24 h incubation. Standardised methodologies for MIC determination are described in European Committee on Antimicrobial Susceptibility Testing (EUCAST) and Clinical and Laboratory Standards Institute (CLSI) [7] guidelines [VET01-A4 (formerly M31-A3)]. These ensure reproducibility between individual analysts, between laboratories and across both geographical regions and time [5, 8]. CLSI and EUCAST methods require the use of broths formulated to standardises the growth of bacteria of each bacterial species. Thus, the almost universal use of internationally recognised guidelines, methods and standards for MIC determination is highly beneficial. However, when the objective of potency determination is prediction of dosage for clinical efficacy based on PK/PD breakpoints, conditions should be representative of in vivo pathological circumstances. Zeitlinger et al. [9] commented that “bacteria with appropriate and well-defined growth in the selected medium should be employed” and “in order to be able to extrapolate data from various models to in vivo situations, models should always attempt to mimic physiological conditions as closely as possible”.

Whilst serum is not identical to extravascular infection site fluids, it is likely to be closer to the biophase than broths in chemical composition and indeed in respect of immunological components also [9, 10]. A comparison of broth MICs with potency determined in biological fluids is therefore relevant to PK/PD breakpoint estimation, when the aim is optimal dose prediction for clinical use. For some drugs and pathogens, calculation of a scaling factor to bridge between broth and serum MICs may be warranted [4, 6, 10].

A second consideration, in relation to dose prediction, is that CLSI and EUCAST guidelines have limitations regarding accuracy for individual isolates, because they require use of two-fold dilutions, giving potential for approaching 100% error. For most purposes, this is wholly acceptable, and indeed is necessary, when large numbers of isolates are examined to establish MIC distributions. When plotted on a histogram, using a log-base 2 distribution, they are log-normal. These histograms facilitate the identification of wild-type distributions. Standardisation of methodology thereby enables determination of epidemiological cut-offs (ECOFF) by EUCAST and wild type cut-offs (CO_WT_) by CLSI. However, modification of the two-fold dilution approach may be appropriate, when MICs are correlated with PK data for establishing PK/PD breakpoints. Based on methods previously described [11, 12] five overlapping sets of doubling dilutions were used in this study to decrease inaccuracy from up to 100% to no more than 20% for a small number of individual isolates. Hence, the quantitative determination potency with improved accuracy and in biological fluids may be appropriate for some drug classes and some microorganisms, when calculating PK/PD indices [6, 10, 12–16].

It is the hypothesis of this investigation that MICs determined in pig serum (potentially reflecting more closely concentrations required to achieve clinical efficacy than those determined in artificial broths) might differ from MICs determined in broths. The aims were to: (1) compare MICs measured in five broths with MICs determined in pig serum, using five sets of overlapping two-fold dilutions for three drugs, florfenicol, marbofloxacin and oxytetracycline, used in the treatment of pig pneumonia caused by the pathogens, *Actinobacillus pleuropneumoniae* and *Pasteurella multocida*; (2) determine whether any broth/serum MIC differences were attributable solely to drug binding to serum protein; (3) compare chemical compositions of CLSI recommended broths and pig serum; (4) determine rates of growth *A. pleuropneumoniae* and *P. multocida* in broths and pig serum in the absence of drugs. Aims 3 and 4 were included to investigate whether chemical composition differences and/or bacterial growth rate differences between broth and serum might account for matrix MIC differences over and above those attributable binding of the drugs to serum protein.

## Methods

### Selection of bacterial isolates for MIC studies

Don Whitley Scientific (Shipley, West Yorkshire, UK) supplied 20 isolates of *P. multocida* and three American Type Culture Collection (ATCC) reference strains to validate MIC tests; *A. pleuropneumoniae* ATCC 27090, *Enterococcus faecalis* ATCC 29212 and *Escherichia coli* ATCC 25922. Eight isolates of *A. pleuropneumoniae* were supplied by A. Rycroft (Royal Veterinary College, Hatfield, Herts, UK). All *P. multocida* and *A. pleuropneumoniae* isolates were obtained from European Union (EU) field cases of pig pneumonia, specifically the UK and France. The quality control organisms were incorporated in each MIC test. Organisms were stored at -80 °C.

From the organisms supplied, three isolates of each species were selected, based on three criteria: (1) exhibiting logarithmic growth in all broths (see below) and pig serum; (2) sensitivity to florfenicol, marbofloxacin and oxytetracycline in disk diffusion assays (data not shown); and (3) the highest and lowest CLSI defined broth MICs and one isolate of intermediate MIC, determined using two-fold dilutions (data not shown).

### Culture media and bacterial counts

For *A. pleuropneumoniae* Chocolate Mueller Hinton Agar (CMHA) was used to grow the organism on a solid medium. Mueller Hinton Agar (MHA), supplemented with 5% defibrinated sheep blood, was used to grow *P. multocida*. Columbia broth (CB) supplemented with 2 μg/mL nicotinamide adenine dinucleotide (NAD) was used as the principal liquid broth for *A. pleuropneumoniae* MIC determination. CLSI guidelines require use of Veterinary Fastidious Medium (VFM) for the liquid culture of *A. pleuropneumoniae*. However, for isolates used in this study, MIC end-points were more readily and reliably established using CB than with VFM. In the latter broth, there was difficulty defining MIC end-points, because the high density of red blood cells caused them to settle at the base of the well obscuring the result of MIC. The CLSI recommended broth for *P. multocida* is Cation Adjusted Mueller Hinton broth (CAMHB), and this was the principal liquid broth used in this study for this organism.

Both organisms were incubated in a static incubator at 37 °C for 18–24 h. For comparative purposes, MICs were determined for both organisms in five broths, CB, CAMHB, Mueller Hinton broth (MHB), Brain Heart Infusion broth (BHI), Tryptic soy broth (TSB), and pig serum (Invitrogen Gibco Porcine Serum, Origin: New Zealand, ThermoFisher Scientific, UK).

Bacterial counts were determined by serial dilution and spot plate counts. Culture dilutions were carried out in phosphate buffered saline. Three 10 μL drops of the appropriate dilutions were dropped onto the agar surface and allowed to dry. After 24 h incubation, the mean colony forming unit (CFU) count for each 10 μL was determined and multiplied by 100 and then multiplied by the dilution factor to give the original CFU/mL count.

### Minimum inhibitory concentrations in six growth matrices

MICs of florfenicol, marbofloxacin and oxytetracycline were determined by microdilution in 96-well plates for three isolates each of *A. pleuropneumoniae* and *P. multocida*, using CLSI guidelines*,* except that: (1) five sets of overlapping two-fold serial dilutions were used to reduce inaccuracy for individual isolates to no greater than 20%; (2) determinations were made in five broths, MHB, CAMHB, CB, BHIB, TSB and pig serum; (3) the bacterial culture was grown to 0.5 McFarland Standard and this was diluted ten-fold to obtain the intended starting inoculum of 1–2 × 10^7^ CFU/mL. This starting inoculum count is higher than the CLSI recommendation of 5 × 10^5^ CFU/mL, the higher inoculum count being selected deliberately to be equivalent clinically to a medium to heavy in vivo microbial challenge.

Drug solutions, media and culture were added sequentially to the wells of 96-well plates, with a total volume of 200 μL. These were sealed and incubated statically at 37 °C for 24 h. Spot plate counts were prepared immediately after plate inoculation. ATCC isolates were used in all assays at the CLSI recommended strength of 5 × 10^5^ CFU/mL. A positive control well contained medium and pathogen only and a negative control well contained medium and drug only. Blank controls contained medium alone.

MICs were determined in triplicate for each isolate of each species and each drug. In previous studies in this laboratory, binding to pig serum protein had been determined for each drug over the therapeutic concentration range and shown to be independent of concentration [17–19]. Percentage binding was 49, 65 and 71 for marbofloxacin, florfenicol and oxytetracycline, respectively. As serum protein bound drug is microbiologically inactive, MICs for serum were reported first, as uncorrected, experimental values and second, as values corrected for free drug (that is unbound) concentration, termed the fu serum MIC.

### Chemical composition of growth matrices

For analysis of 14 chemical constituents, three batches of each broth were prepared in 10 mL quantities, according to manufacturer’s instructions and three batches each of pig and calf serum were obtained commercially (ThermoFisher Scientific, Paisley, UK). Calf serum was included to compare the composition of pig serum with another farm animal species. Samples were analysed for total protein, albumin, globulin, sodium, potassium, calcium, magnesium, chloride, inorganic phosphorus, urea, creatinine, glucose, iron and Unsaturated Iron Binding Capacity (UIBC) (ILAB 600 Instrumentation Laboratory Ltd., Cheshire UK; Nova Biomedical CCX and Phox, Cheshire UK).

### Bacterial growth in broth and serum

Twenty isolates each of *P. multocida* and *A. pleuropneumoniae* (Don Whitley Scientific (Shipley, West Yorkshire, UK) supplied 20 isolates of both organisms for the purpose of determining growth in media), from clinical cases of pig pneumonia, were used to investigate bacterial growth in the absence of antimicrobial drugs. Growth rates were determined in pig serum and the CLSI recommended broths, VFM for *A. pleuropneumoniae* and CAMHB for *P. multocida*.

Three mL of serum, CAMHB or VFM were aseptically added to the wells of a 96-well plate. Two control wells containing medium alone ensured the absence of contamination. Six independent colonies were removed from a fresh agar plate aseptically and mixed into the medium within the wells. The well plates were placed in a static incubator at 37 °C for 24 h. At times of 0, 6 and 24 h viable counts were determined. Bacterial growth was monitored in triplicate for each of 20 isolates of each species.

## Results

### Minimum inhibitory concentration

Tables 1, 2 and 3 present MIC data for each drug as geometric means and standard deviation for pig serum, MHB, CAMHB, CB, BHIB and TSB.

**Table 1:** *P. multocida* (PM) and *A. pleuropneumoniae* (APP) Minimum Inhibitory Concentrations for marbofloxacin; mean and standard deviation (SD) in pig serum and five broths^a^

	PM (*n* = 3)		APP (*n* = 3)	
Matrix	Mean	SD	Mean	SD
Serum	0.09	0.52	0.69	0.23
fu Serum	0.04	0.27	0.35	0.12
MHB	0.06	0.01	0.93	0.17
CAMHB	0.07	0.05	0.91	0.13
CB	0.25	0.14	1.00	0.00
BHIB	0.27	0.18	1.14	0.25
TSB	0.05	0.01	1.37	0.22

^a^Cation Adjusted Mueller Hinton Broth (CAMHB), Mueller Hinton Broth (MHB), Columbia Broth supplemented with NAD (CB), Brain Heart Infusion broth (BHI) and Tryptic Soy broth (TSB)

**Table 2:** *P. multocida* (PM) and *A. pleuropneumoniae* (APP) Minimum Inhibitory Concentrations for florfenicol; mean and standard deviation (SD) in pig serum and five broths^a^

	PM (*n* = 3)		APP (*n* = 3)	
Matrix	Mean	SD	Mean	SD
Serum	0.30	0.13	0.47	0.18
fu Serum	0.10	0.05	0.16	0.06
MHB	0.47	0.03	0.36	0.02
CAMHB	0.55	1.19	0.36	0.04
CB	0.50	0.00	0.43	0.03
BHI	0.50	0.00	0.45	0.05
TSB	0.49	0.02	0.44	0.04

^a^Cation-adjusted Mueller Hinton Broth (CAMHB), Mueller Hinton Broth (MHB),, Columbia Broth supplemented with NAD (CB), Brain Heart Infusion broth (BHI) and Tryptic Soy broth (TSB)

**Table 3:** *P. multocida* (PM) and *A. pleuropneumoniae* (APP) Minimum Inhibitory Concentrations for oxytetracycline; mean and standard deviation (SD) in pig serum and five broths^a^

	PM (*n* = 3)		APP (*n* = 3)	
Matrix	Mean	SD	Mean	SD
Serum	6.20	2.06	3.47	2.38
fu Serum	1.80	0.60	1.01	0.69
MHB	0.28	0.04	2.56	0.29
CAMHB	0.29	0.04	2.34	0.24
CB	0.30	0.04	2.92	0.28
BHI	0.34	0.05	3.06	0.21
TSB	0.38	0.03	3.22	0.42

^a^Cation-adjusted Mueller Hinton Broth (CAMHB), Mueller Hinton Broth (MHB),, Columbia Broth supplemented with NAD (CB), Brain Heart Infusion broth (BHI) and Tryptic Soy broth (TSB)

### Marbofloxacin

*P. multocida* MICs were similar for three broths, MHB, CAMHB and TSB (range = 0.05–0.07 μg/mL). MICs were 4–5-fold higher for CB and BHIB (0.25–0.27 μg/mL). Serum MIC was 0.09 μg/mL and, corrected for protein binding, fu serum MIC was 0.04 μg/mL. The mean MIC ratio fu serum:CAMHB was 0.63:1.

For *A. pleuropneumoniae* broth MICs were similar (range = 0.91 μg/mL for CAMHB to 1.37 μg/mL for TSB). Serum MIC was 0.69 μg/mL and fu serum MIC was 0.35 μg/mL; mean MIC ratio fu serum:CB was 0.35:1.

### Florfenicol

For *P. multocida* MICs in all broths were similar; range = 0.47 μg/mL for MHB to 0.55 μg/mL for CAMHB. Serum MIC was 0.30 μg/mL and fu serum MIC was 0.10 μg/mL. The mean MIC ratio fu serum:CAMHB was 0.19:1.

For *A. pleuropneumoniae* broth MICs ranged from 0.36 μg/mL for both MHB and CAMHB to 0.45 μg/mL for BHIB. Serum MIC was 0.47 μg/mL and fu serum MIC was 0.16 μg/mL, so that mean MIC ratio fu serum:CB was 0.38:1.

### Oxytetracycline

For *P. multocida* broth MICs were similar, ranging from 0.28 μg/mL (MHB) to 0.38 μg/mL (TSB). Serum MIC was 6.20 μg/mL and fu serum MIC was 1.80 μg/mL. The mean MIC ratio fu serum:CAMHB was 6.30:1.

Broth MICs for *A. pleuropneumoniae* ranged from 2.34 μg/mL (CAMHB) to 3.22 μg/mL (TSB). Serum MIC and fu serum MIC were 3.47 and 1.01 μg/mL, respectively, so that the mean MIC ratio fu serum:CB was 0.35:1.

### Chemical composition of broths and serum

**Fig. 1:**
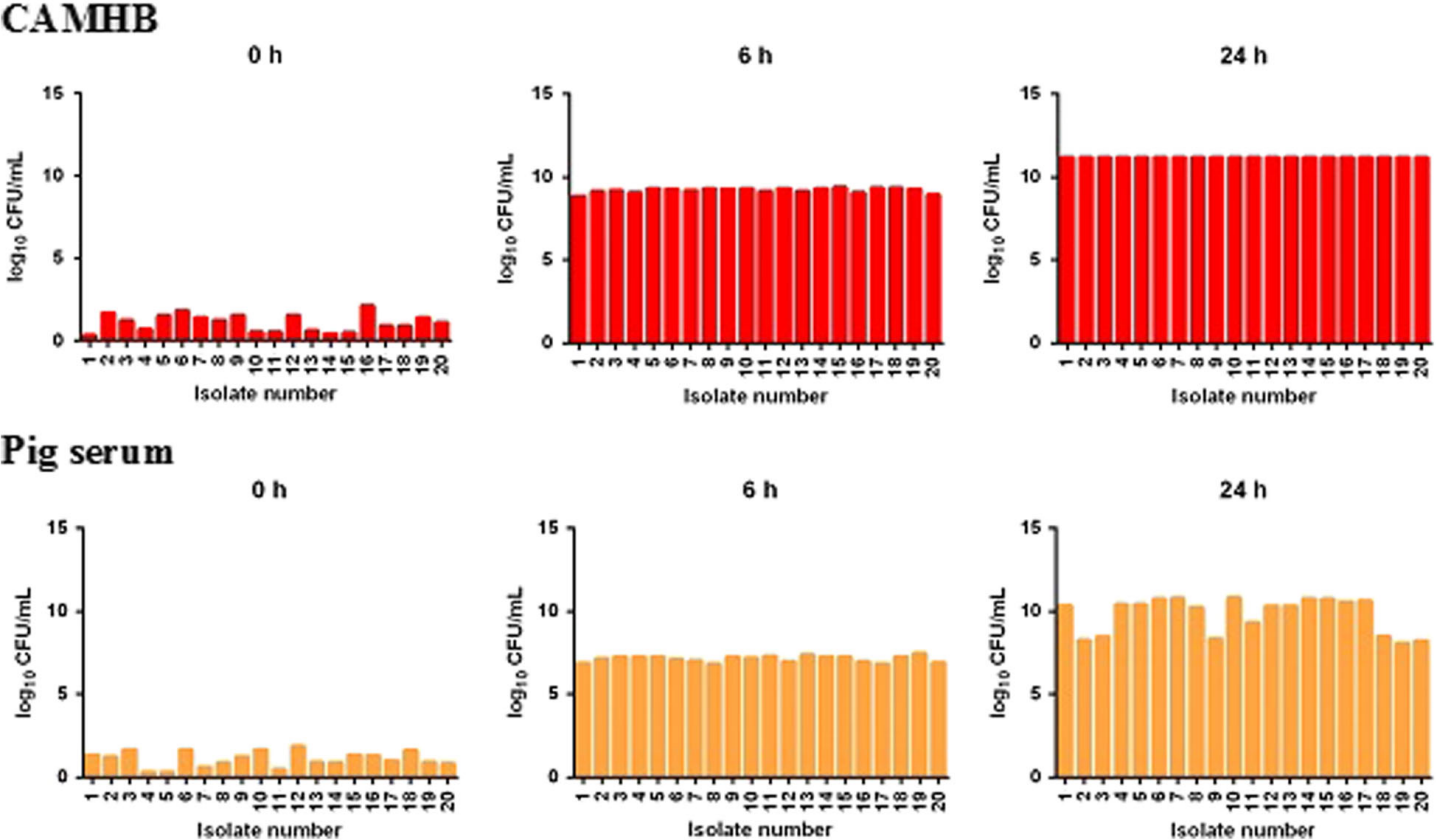
Pie chart comparing the concentrations of constituents in eight matrices: Porcine serum, bovine serum, cation-adjusted Mueller Hinton Broth (CAMHB), Mueller Hinton Broth (MHB), Columbia broth supplemented with NAD (CB), Veterinary Fastidious Medium (VFM), Trypic Soy broth (TSB) and Brain Heart Infusion broth (BHI). Highlighted with a star is one of the more notably important components, total protein

Table 4 presents comparative data on the chemical composition of six broths and pig serum for 14 constituents. In addition, for comparison with the latter, the composition of serum for another farm animal species, the calf, was determined (Fig. 1). The data were derived for the mean of three batch samples of each fluid.

**Table 4:** Chemical composition of broths^a^ and pig and calf sera: mean and standard deviation (SD) (*n* = 3 batches)^a^

Matrix	Total protein (g/L)	Albumin (g/L)	Globulin (g/L)	Sodium (mmol/L)	Potassium (mmol/L)	Calcium (mmol/L)	Magnesium (mmol/L)	Chloride (mmol/L)	Inorganic phosphorus (mmol/L)	Urea (mmol/L)	Creatinine (μmol/L)	Glucose (mmol/L)	Iron (μmol/L)	Unsaturated Iron Binding Capacity (μmol/L)
Porcine serum	79.3	40.3	39.0	153	7.13	2.59	0.98	115	2.82	4.67	139	5.47	19.1	78.00
SD	2.20	0.17	2.03	4.4	0.06	0.14	0.09	8.96	0.61	3.35	5.5	0.84	0.15	0.36
Bovine serum	74.3	38.1	37.1	148	7.07	2.55	1.00	108	3.28	5.23	149	4.43	22.2	71.00
SD	3.65	2.11	1.31	15.4	1.04	0.55	0.17	7.55	0.98	0.60	9.9	0.29	0.61	0.43
MHB	3.23	0.00	3.23	125	2.49	0.13	0.23	99.23	2.55	0.93	61.3	2.57	9.47	-6.90
SD	0.12	0.00	0.12	0.2	0.01	0.02	0.00	0.32	0.03	0.06	2.31	0.06	0.15	0.44
CAMHB	4.10	0.07	4.03	127	2.87	5.21	4.20	117	2.96	2.97	83.3	2.57	9.53	-7.20
SD	0.44	0.06	0.46	11.5	0.21	2.14	1.68	5.29	0.34	0.64	2.08	0.25	0.17	1.24
CB	12.6	0.10	12.5	130	14.58	0.22	1.41	112	4.99	2.90	217	12.90	63.9	-56.50
SD	0.20	0.00	0.20	0.1	0.01	0.01	0.02	0.26	0.02	0.10	2.31	0.00	0.62	1.74
BHI	9.67	0.10	9.57	168	10.01	0.13	0.25	123	6.86	5.97	418	10.20	7.50	-3.17
SD	0.21	0.00	0.21	0.2	0.01	0.01	0.01	0.56	0.07	0.06	3.61	0.20	0.10	1.11
TSB	9.07	0.10	9.00	118	9.56	0.05	0.18	79.33	4.85	2.17	260	11.77	6.86	-2.20
SD	0.06	0.06	0.10	0.1	0.01	0.02	0.00	0.06	0.02	0.06	1.53	0.15	0.25	1.74
VFM	11.4	0.87	10.5	132	38.73	0.33	1.16	104	14.31	4.87	198	3.40	25.9	-3.00
SD	0.26	0.12	0.15	3.5	0.31	0.03	0.10	4.04	0.44	0.72	9.07	0.10	0.52	1.31

^a^Mueller Hinton Broth (MHB), Cation Adjusted Mueller Hinton Broth (CAMHB),, Veterinary Fastidious Medium (VFM), Columbia Broth supplemented with NAD (CB), Brain Heart Infusion broth (BHIB), Tryptic Soy broth (TSB) and Veterinary Fastidious Medium (VFM)

### Protein, albumin and globulin

For the six broths, total protein concentration was low in MHB and CAMHB (3.23 and 4.10 g/L, respectively) but higher and similar for the four other broths (range 9.07–12.6 g/L). Serum total protein concentrations were higher, 79.3 g/L (pig) and 74.3 g/L (calf). For all broths, the principal protein constituent was globulin (range 3.23 to 12.5 g/L) whilst albumin concentrations were uniformly low, ranging from 0.00 to 0.87 g/L, for MHB and VFM, respectively. Albumin serum concentrations were much higher; 40.2 g/L (pig) and 38.1 g/L (calf). Expressed as serum:broth albumin concentration ratios, these ranged from greater than 400:1 for CAMHB to 46:1 for VFM.

### Electrolytes

There were moderate differences in sodium concentration between the growth matrices; the lowest concentration was 118 mmol/L (TSB) and the highest was 168 mmol/L (BHI) and serum concentrations were intermediate, 153 and 148 mmol/L, for pig and calf, respectively. Similarly, serum chloride ion concentrations for pig and calf were intermediate (115 and 108 mmol/L, respectively) between the lowest, 79 mmol/L for TSB, and highest broth concentration of 123 mmol/L for BHI. MHB had the lowest potassium concentration (2.49 mmol/L) whereas the highest concentration was 39 mmol/L for VFM. Serum potassium concentrations were intermediate, 7.13 and 7.07 mmol/L for pig and calf, respectively.

With the exception of CAMHB, the concentration of calcium was much lower in broths, ranging from 0.05 to 0.33 mmol/L, in comparison with serum, 2.59 and 2.55 mmol/L for pig and calf, respectively. For CAMHB the calcium concentration was 5.21 mmol/L. Concentrations of magnesium were lower in three broths (0.23 mmol/L MHB, 0.25 mmol/L BHI and 0.18 mmol/L TSB) than in serum of pig and calf, 0.98 and 1.00 mmol/L, respectively, whereas concentrations in CAMHB, CB and VFM were higher, 4.20, 1.41 and 1.16, respectively.

### Inorganic phosphorus, urea, creatinine and glucose

Broth inorganic phosphorus concentrations ranged from 2.55 mmol/L (MHB) to 14.3 mmol/L (VFM) and serum concentrations were similar to MHB, 2.82 and 3.28 mmol/L for pig and calf, respectively. Broth urea concentrations ranged from 0.93 mmol/L (MHB) to 5.97 mmol/L (BHI) and concentrations in serum were slightly less than the highest broth value, 4.67 and 5.23 mmol/L, pig and calf, respectively. In broths, creatinine concentration was lowest in MHB (61 μmol/L) and highest in BHI (418 μmol/L) and serum concentrations were intermediate (139 and 149 μmol/L, for pig and calf). Broth glucose concentrations ranged from 2.56 mmol/L (MHB and CAMHB) to 12.9 mmol/L (CB) whilst serum concentrations were intermediate, 5.47 mmol/L (pig) and 4.43 mmol/L (calf).

### Iron and unsaturated iron binding capacity

Iron broth concentrations varied almost 10-fold, from 6.86 μmol/L (TSB) to 63.9 μmol/L (CB) and serum concentrations were intermediate, 19.1 μmol/L (pig) and 22.2 μmol/L (calf). Unsaturated iron binding capacity also varied widely (data not shown).

### Bacterial growth in absence of antimicrobial drugs

**Fig. 2:**
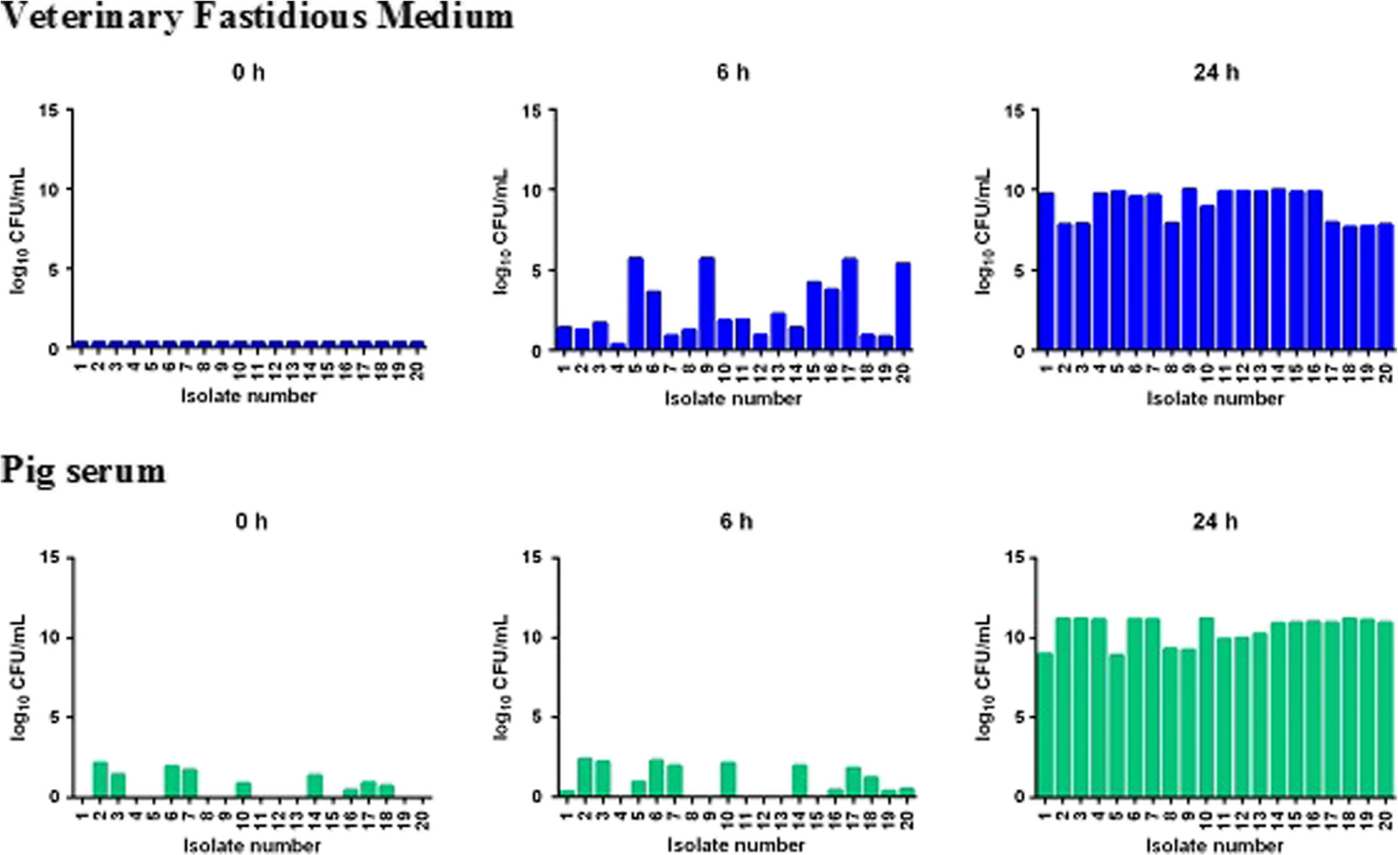
In vitro growth of *P. multocida* (*n* = 20 isolates) at time points 0, 6 and 24 h in CAMHB and pig serum (*n* = 20). Growth quantified in log_10_ CFU/mL (ordinate); each bar represents mean of triplicate analyses

**Fig. 3:**
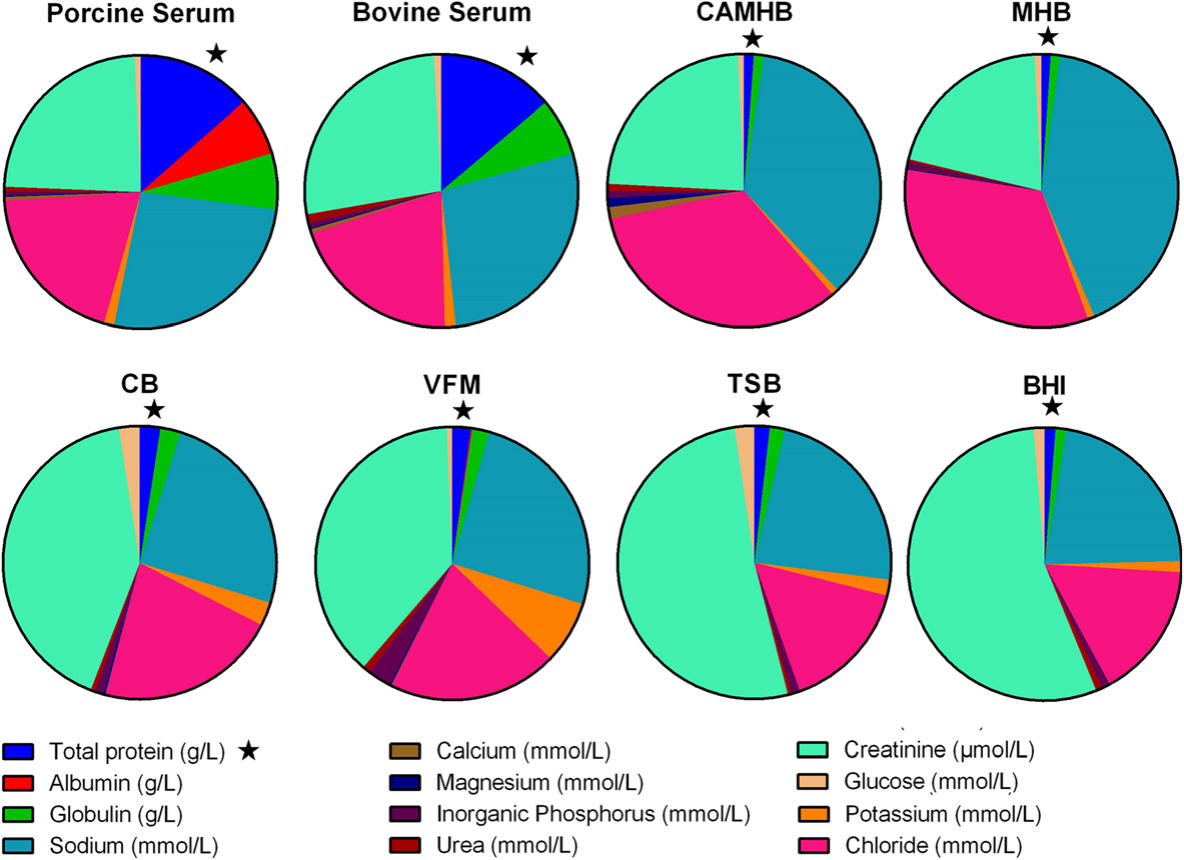
In vitro growth of *A. pleuropneumoniae* (*n* = 20 isolates) at time points 0, 6 and 24 h in VFM and pig serum. Growth quantified in log_10_ CFU/mL (ordinate); each bar represents mean of triplicate analyses

Figures 2 and 3 illustrate bacterial growth rates of *P. multocida* and *A. pleuropneumoniae*, respectively. There were no apparent inter-isolate differences in *P. multocida* growth rates in CAMHB at 6 and 24 h. For all isolates at 6 h, bacterial counts had increased to 6-7x10^9^CFU/mL in broth and at 24 h counts exceeded 10^10^ CFU/mL for all isolates. In serum, there was again minimal inter-isolate difference in growth of *P. multocida* at 6 h but the count, 5-6x10^7^CFU/mL, was lower at this time than in CAMHB by some 2 × 10^2^ CFU/mL. After 24 h, there was further increase in growth but there were some inter-isolate differences in count, which ranged from 8-10 × 10^10^ CFU/mL, and the serum count was somewhat lower than the 24 h count in CAMHB at this time.

For *A. pleuropneumoniae,* bacterial growth was much slower initially (at 6 h) in both media than for *P. multocida.* At 6 h the mean CFU/mL count for 20 isolates was 5.37 × 10^5^ in broth and in in serum it was significantly lower, 1.36x10^2^CFU/mL. In contrast with *P. multocida* there was clear inter-isolate variability in growth rate at 6 h. However, by 24 h considerable growth had occurred and counts were of a similar order in both media, approaching or exceeding slightly 10log_10_ CFU/mL.

## Discussion

For some drug classes, MICs measured in broths using internationally standardised procedures (for example, those of CLSI and EUCAST) may not differ significantly from potency estimated in biological fluids. The absence of growth matrix differences implies no significant impact on PK/PD breakpoint estimation, as a basis for dose determination, provided that the protein bound serum drug concentration is corrected for [4, 16, 20]. However, broth/biological fluid differences in potency do occur for other drug classes, even after correction for drug binding to proteins in biological fluids. For example, Honeyman et al. [21] compared potencies of several tetracyclines in broth and a 50% broth: 50% serum mixture. They established marked differences in MIC for the two growth matrices. Moreover, MICs differed and had to be determined on a matrix-by-matrix, species-by-species, compound-by-compound basis. Likewise, Brentnall et al. [10, 14] reported for oxytetracycline, after correction for serum protein binding, for a calf isolate of *Mannheimia haemolytica*, a MIC in serum 6 times *greater* than the broth MIC. In marked contrast, Toutain et al. [6] reported, after correction for serum protein binding, MICs some 80-fold *smaller* in calf serum compared to broth for tulathromycin for *M. haemolytica* and *P. multocida* isolates from calves. Similar findings, with quantitatively even lower MICs for *Mycoplasma mycoides mycoides* in calf serum compared to broth, were reported for the macrolides, tulathromycin and gamithromycin by Mitchell et al. [22–24]. Thus, whilst correction for serum protein binding is always necessary, it is not always sufficient to account for potency differences between matrices.

These published data suggest: first, that serum MIC values should be considered on both a drug-by-drug and bacterial species-by-species basis to allow for the inactive protein bound fraction; and second, corrected serum values may not be the same as, and therefore might be used in preference to, the broth MIC for PK/PD breakpoint estimation.

Only free drug is microbiologically active and therefore protein binding is a major factor, and unfortunately sometimes the only factor, considered in seeking to explain growth medium differences in antimicrobial drug potency [2, 3, 9, 16, 20, 25–27]. The magnitude of drug binding to serum protein can vary with methodology [16] and, moreover, for single drugs, intra-species differences have been reported. In addition, it is necessary to consider possible differences in protein binding in serum obtained from different sources, differing animal breeds, ages and indeed between healthy and diseased animals. Such variations must be borne in mind in considering the present findings, for which protein binding was determined for pig serum from a single source in healthy animals. In fact, for all three drugs investigated, the degree of binding was independent of total concentration over therapeutic ranges [17–19].

For both organisms investigated in this study, correcting the serum MIC for protein binding for marbofloxacin, florfenicol and oxytetracycline yielded fu serum MICs differing from broth MICs in most instances. Fu serum:broth MIC ratios were: marbofloxacin 0.63:1 (*P. multocida*) and 0.35:1 (*A. pleuropneumoniae*); florfenicol 0.19:1 (*P. multocida*) and 0.38:1 (*A. pleuropneumoniae*); oxytetracycline 6.30:1 (*P. multocida*) and 0.35:1 (*A. pleuropneumoniae*)*.* Thus, for marbofloxacin and florfenicol there were small to moderate trends for both pathogens of increased (1.6- to 5.2-fold) potency in serum compared to broth. Likewise, for oxytetracycline and *A. pleuropneumoniae* the greater potency in serum was 2.8-fold, whereas for *P. multocida* potency was 6.3-fold lower in serum. Consequently, correction of serum MIC for protein binding is, as stated, *necessary but not sufficient* for determination of potency differences between the two matrices for these two bacterial species and these three drugs. The consistent finding of differences, which were nevertheless unpredictable in direction (increased or reduced potency) and magnitude indicates the possibility of similar differences for human pathogens also more frequently than is commonly recognised.

Possibly the most important difference in chemical composition between all broths and serum was in albumin concentration, which was much higher in serum. Correction for drug binding to serum albumin revealed broth/serum potency differences for all drugs. It is therefore necessary to consider whether other differences, as well as albumin content, in chemical composition might explain the protein binding corrected potency differences. The chemical analyses indicated wide differences in composition of five broths for electrolytes, iron and organic compounds. Possibly surprisingly, these frequently large inter-broth differences resulted in, at most, very minor differences in MICs, with one exception, namely marbofloxacin, and for *P. multocida* only. However, even for this drug and species, MICs were similar for two broths and different but again similar for other three broths. In contrast, the broth and corrected serum MICs differed, despite the finding that chemical composition of pig serum for every analyte was within the lower and upper ranges for the five broths. Therefore, chemical composition indicated no readily apparent explanation for the broth/serum MIC differences.

In addition to the chemical differences between broths and serum, for circulating blood there are other differences e.g. the presence of white and red cells as well as a wide range of immunological components in blood. Future studies should therefore be directed towards evaluating which of these might interact with drugs, and how, to modulate drug potency. One factor, pH, is known to influence the rate and extent of growth of microorganisms [28, 29]. An effect of pH on weakly basic drugs such as macrolides and triamilides is well recognised [6].

Zeitlinger et al. [26] compared growth curves of *Staphylococcus aureus* and *Pseudomonas aeruginosa* in MHB and serum. Slower logarithmic growth was obtained for both species in serum compared to broth, and this might be expected to provide more rapid kill in serum, as a consequence of a smaller microbial challenge [5, 30]. Indeed, Illambas et al. [31, 29] reported significant effects of inoculum count (high, medium and low) on MIC for marbofloxacin and florfenicol and the calf pneumonia pathogens, *M. haemolytica* and *P. multocida*, while Dorey et al. [17–19] reported similar dependency of MIC on inoculum strength for the three drugs and the two pig pathogens investigated in this study.

In the present study, bacterial growth was initially greater in broths than serum for both *P. multocida* and *A. pleuropneumoniae*. This difference persisted for *P. multocida* at 24 h, whereas for *A. pleuropneumoniae* logarithmic growth was similar for the two media at this time. Slower growth, resulting potentially in lesser challenge to bacterial kill, therefore might explain, at least in part, the greater potency in serum compared to broth in five of six instances. The one notable exception, however, was the 6-fold reduction in potency of oxytetracycline in serum compared to broth for *P. multocida.* This finding indicates that some serum factor, presently unknown, operates to reduce potency of this drug for this species, whilst conversely potency was increased in serum for *A. pleuropneumoniae.*

The differences in MIC between serum and broth reported in this study do not provide a rationale for abandoning broths by diagnostic laboratories reporting MIC distributions of wild type organisms. This would be impractical and unnecessary. Rather, the present data suggest that, for marbofloxacin, florfenicol and oxytetracycline and the two bacterial species studied, it will be appropriate and possible to apply a scaling factor, to bridge between MICs in broths and pig serum when calculating PK/PD breakpoints.

## Conclusion

For three commonly used drugs, florfenicol, oxytetracycline and marbofloxacin, and two pathogenic species of bacteria causing pig pneumonia, *A. pleuropneumoniae* and *P. multocida*, differences between serum and broth MICs were obtained, after correction was made for binding to serum protein. In contrast, major differences were not obtained when MICs were determined in five broths, marked differences in chemical composition between the broths notwithstanding. In the absence of drugs, the rate of logarithmic growth of *A. pleuropneumoniae* and *P. multocida* was slower in pig serum than in broth. It is suggested that, in using MIC data as a pharmacodynamic parameter to determine PK/PD breakpoints, the use of a scaling factor to bridge between broth and biological fluids may be required more commonly than currently recognised.

## Highlights

- For florfenicol, oxytetracycline and marbofloxacin and the pig pathogens, A. pleuropneumoniae and P. multocida, there were no major differences in MICs measured in five broths, widely differing in chemical composition.
- Significant differences between serum and broth MICs were obtained, after correction for binding to serum protein, indicating that such correction is necessary but that it does not alone account for potency differences.
- With one exception potency was greater in serum than broths.
- In using MIC data to predict dosages for clinical use, the use of a scaling factor to bridge between broth and biological fluids may be required.

## Abbreviations

ATCC: American Type Culture Collection
BHI: Brain Heart Infusion Broth
CAMHB: cation-adjusted Mueller Hinton Broth
CB: Columbia Broth
CLSI: Clinical and Laboratory Standards Institute
EUCAST: Committee on Antimicrobial Susceptibility Testing
MHB: Mueller Hinton Broth
MIC: Minimum inhibitory concentration
NAD: Nicotinamide Adenine Dinucleotide
PD: Pharmacodynamics
PK: Pharmacokinetics
TSB: Tryptic Soy Broth
VFM: Veterinary Fastidious Medium

## References

[1] Toutain PL, Lees P (2004). Integration and modelling of pharmacokinetic and pharmacodynamic data to optimize dosage regimens in veterinary medicine. J Vet Pharmacol Ther.

[2] Nielsen EI, Cars O, Friberg LE (2011). Pharmacokinetic/pharmacodynamic (PK/PD) indices of antibiotic predicted by a semimechanistic PK/PD model: a step toward model-based dose optimization. Antimicrob Agents Chemother.

[3] Nielsen EI, Friberg LE (2013). Pharmacokinetic-Pharmacodynamic modeling of antibacterial drugs. Pharmacol Rev.

[4] Lees P, Pelligand L, Illambas J, Potter T, Lacroix M, Rycroft A (2015). Pharmacokinetic/pharmacodynamic integration and modelling of amoxicillin for the calf pathogens Mannheimia haemolytica and Pasteurella multocida. J Vet Pharmacol Ther.

[5] Martinez MN, Papich MG, Drusano GL (2012). Dosing regimen matters: the importance of early intervention and rapid attainment of the pharmacokinetic/pharmacodynamic target. Antimicrob Agents Chemother.

[6] Toutain PL, Potter T, Pelligand L, Lacroix M, Illambas J, et al. Standard PK/PD concepts can be applied to determine a dosage regimen for a macrolide: the case of tulathromycin in the calf. J Vet Pharmacol Ther 2016; doi: 10.1111/jvp.12333, in press. 10.1111/jvp.12333 27501187

[7] CLSI. Performance Standards for Antimicrobial Disk and Dilution Susceptibility Tests for Bacteria Isolated from Animals: Approved Standard - Fourth Edition. CLSI document VET01-A4 (formerly M31-A3, 2008) Supplementary information VET01-S, 2015. ISBN 1–56238–877-0 [print]; ISBN 1–56238–878-9 [electronic]. Clinical and Laboratory Standards Institute, 950 West Valley Road, Suite 2500, Wayne, Pennsylvania 19087 USA, 2013.

[8] Papich MG (2014). Pharmacokinetic-Pharmacodynamic (PK-PD) modelling and the rational selection of dosage regimes for the prudent use of antimicrobial drugs. Vet Micro.

[9] Zeitlinger MA, Derendorf H, Mouton JW, Cars O, Craig WA (2011). Protein Binding: Do We Ever Learn?. Antimicrob Agents Chemother.

[10] Brentnall C, Cheng Z, McKellar QA, Lees P (2012). Pharmacodynamics of oxytetracycline administered alone and in combination with carprofen in calves. Vet Rec.

[11] Aliabadi FS, Lees P (2001). Pharmacokinetics and pharmacodynamics of danofloxacin in serum and tissue fluids of goats following intravenous and intramuscular administration. Amer J Vet Res.

[12] Sidhu P, Landoni MF, Aliabadi FS, Lees P (2010). Pharmacokinetic and pharmacodynamic modelling of marbofloxacin administered alone and in combination with tolfenamic acid in goats. Vet J.

[13] Aliabadi FS, Lees P (2002). Pharmacokinetics and pharmacokinetic/pharmacodynamic integration of marbofloxacin in calf serum, exudate and transudate. J Vet Pharmacol Ther.

[14] Brentnall C, Cheng Z, McKellar QA, Lees P (2013). Pharmacokinetic-pharmacodynamic integration and modelling of oxytetracycline administered alone and in combination with carprofen in calves. Res Vet Sci.

[15] Nightingale CH, Murakawa T. Microbiology and pharmacokinetics. In Microbiology and pharmacokinetics Eds Nightingale, C.H., Murakawa, T. Ambrose, P.G. Marcel Dekker, A.G. New York: Marcel Dekker, Inc.; 2002. pp. 23–39.

[16] Barbour AM, Schmidt S, Zhuang L (2014). Application of pharmacokinetic/pharmacodynamic modelling and simulation for the prediction of target attainment of ceftobiprole against meticillin-resistant *Staphylococcus aureus* using minimum inhibitory concentration and time–kill curve based approaches. Int J Antimicrob A.

[17] Dorey L, Hobson S, Lees P. What is the true in vitro potency of oxytetracycline for the pig pneumonia pathogens A. pleuropneumoniae and P. multocida ? J Vet Pharmacol Ther. 2016. doi:10.1111/jvp.12386. 10.1111/jvp.12386 PMC5600113 28101885

[18] Dorey L, Hobson S, Lees P. Activity of florfenicol for the porcine pneumonia pathogens Actinobacillus pleuropneumoniae and Pasteurella multocida using standardised versus non-standardised methodology. Vet J. 2016; doi:10.1016/j.tvjl.2016.11.004. 10.1016/j.tvjl.2016.11.004 27938711

[19] Dorey L, Hobson S, Lees P. Potency of marbofloxacin for pig pneumonia pathogens Actinobacillus pleuropneumoniae and Pasteurella multocida: comparison of growth media. Res Vet Sci. 2016; doi:10.1016/j.rvsc.2016.11.001. 10.1016/j.rvsc.2016.11.001 27940285

[20] Gonzalez D, Schmidt S, Derendorf H (2013). Importance of relating efficacy measures to unbound drug concentrations for anti-infective Agents. Clin Microbiol Rev.

[21] Honeyman L, Ismail M, Nelson ML, Bhatia B, Bowser TE, Chen J (2015). Structure-activity relationship of the aminomethylcyclines and the discovery of omadacycline. Antimicrob Agents Chemother.

[22] Mitchell JD, McKellar QA, McKeever DJ. Pharmacodynamics of antimicrobials against Mycoplasma mycoides mycoides small colony, the causative agent of contagious bovine pleuropneumonia: PLoS One 2012; v. 7, p. e44158. 10.1371/journal.pone.0044158 PMC3428318 22952911

[23] Mitchell JD, Goh S, McKellar QA, DJ MK (2013). In vitro pharmacodynamics of gamithromycin against Mycoplasma mycoides subspecies mycoides small Colony. Vet J.

[24] Mitchell JD, McKellar QA, McKeever DJ (2013). Evaluation of antimicrobial activity against Mycoplasma mycoides subsp. mycoides small Colony using an in vitro dynamic dilution pharmacokinetic/pharmacodynamic model. J Med Microbiol.

[25] Wise R (1986). The clinical relevance of protein binding and tissue concentrations in antimicrobial therapy. Clin Pharmacokinet.

[26] Zeitlinger MA, Sauermann R, Traunmüller F, Georgopoulos A, Müller M, Joukhadar C (2004). Impact of plasma protein binding on antimicrobial activity using time-killing curves. J Antimicrob Chemother.

[27] Zeitlinger M, Sauermann R, Fille M, Hausdorfer J, Leitner I, Müller M (2008). Plasma protein binding of fluoroquinolones affects antimicrobial activity. J Antimicrob Chemother.

[28] Russell JB, Dombrowski DB (1980). Effect of pH on the efficiency of growth by pure cultures of rumen bacteria in continuous culture. Appl Environ Microbiol.

[29] Illambas J, Potter T, Sidhu P, Rycroft AN, Cheng Z (2013). Pharmacodynamics of florfenicol for calf pneumonia pathogens. Vet Rec.

[30] Drusano GL (2011). What are the properties that make an antibiotic acceptable for therapy of community acquired pneumonia?. J Antimicrob Chemother.

[31] Illambas J, Potter T, Cheng Z, Rycroft A, Fishwick J (2013). Pharmacodynamics of marbofloxacin for calf pneumonia pathogens. Res Vet Sci.

